# Association between serum ferritin and hypertension according to the working type in Korean men: the fifth Korean National Health and nutrition examination survey 2010–2012

**DOI:** 10.1186/s40557-018-0251-y

**Published:** 2018-06-11

**Authors:** Dong-Hoon Lee, Seong-Kyu Kang, Won-Jun Choi, Kyeong Min Kwak, Dukyun Kang, Sang Ha Lee, Jun-Hyung Lee

**Affiliations:** 1 Department of Occupational and Environmental Medicine, Gachon University Gil Medical Center, 21 Namdong-daero 774 beon-gil, Namdong-gu, Incheon, 21565 Republic of Korea; 2 Department of Occupational and Environmental Medicine, College of Medicine, Gachon University, 38-13 Dokjeom-ro 3 beon-gil, Namdongogu, Incheon, 21565 Republic of Korea

**Keywords:** Ferritin, Shift work, Inflammation, Hypertension

## Abstract

**Background:**

Several studies suggest that serum ferritin concentrations reflect systemic inflammation, and high ferritin levels can increase the risk of hypertension in adult men. Shift work is also known to increase the risk of hypertension; however, there has been no study about the relationship between serum ferritin levels and the prevalence of hypertension according to the working type.

**Methods:**

This cross-sectional study included 4,442 male participants (3,651 daytime workers and 791 shift workers) who participated in the fifth Korean National Health and Nutrition Examination Survey. Hypertension was defined as a systolic blood pressure greater than or equal to 140 mmHg, a diastolic blood pressure greater than or equal to 90 mmHg or the current use of antihypertensive medications regardless of blood pressure values. For the statistical analyses, serum ferritin levels were reclassified into quartiles, and complex sample analyses were used to evaluate the relationship between serum ferritin levels and the prevalence of hypertension according to the working type in this study.

**Results:**

Serum ferritin and shift work were positively associated with the prevalence of hypertension. The effect of interaction was above multiplicative. When compared to participants in the lowest serum ferritin quartile, the odds ratio for hypertension for participants in the highest serum ferritin quartile was 1.372 (1.027–1.833) in daytime workers and 2.009 (1.042–3.873) in shift workers after adjustment.

**Conclusions:**

The prevalence of hypertension increased as ferritin levels increased in individuals, especially in shift workers.

## Background

Iron plays an important role in maintaining the body’s physiological homeostasis [1, 2]. Serum ferritin is a sensitive parameter used to assess iron status in the body and is a well-known diagnostic biomarker for iron deficiency [3]. In addition, serum ferritin is known to reflect systemic inflammation as an acute phase reaction, and several studies have shown that inadequately elevated iron stores may adversely affect health outcomes [4–6]. Several studies have shown that elevated serum ferritin levels are associated with insulin resistance and type 2 diabetes [7–9], metabolic syndrome [9–11], dyslipidemia [12], and obesity [13, 14]. The relationship between serum ferritin and hypertension has not been well established in women and has been controversial, but reports have found an association between serum ferritin and hypertension in men [15–18].

According to several studies, results are relatively consistent regarding the relationship between shift work and increased blood pressure [19–21]. The underlying mechanism includes changes in lifestyle factors caused by the disruption of circadian rhythms. This disruption can lead to adverse changes in a person’s life such as higher stress levels including psychosocial, behavioral, and physiological stress. Among the mechanisms of stress come from shift work, physiological stress can induce inflammation [22]. This inflammation plays a critical role in the atherosclerotic process, all stages of atheroma formation, and coronary heart disease [23]. Several studies have explored the association between shift work and the atherosclerotic process. Two studies assessed this association using ultrasound measurements to measure the extent of subclinical atherosclerosis of the carotid intima media. One reported an increased risk of atherosclerosis for shift workers over 45 years of age [24]. The other study focused on younger daytime and shift workers (24–39 years old) but found a similar association in men [25]. A study reported the mechanism to explain the association between shift work and the atherosclerotic process; high sensitive C-reactive protein, high leukocyte and lymphocyte counts, and natural killer cell activity have all been implicated in this process [22].

There has been no study about the relationship between serum ferritin and hypertension according to the working type. This study was based on large-scale national data representative of the population of the Republic of Korea. We conducted this research to investigate the association between serum ferritin levels and hypertension according to the type of work.

## Methods

### Study populations

We used data from the fifth Korean National Health and Nutrition Examination Survey (KNHANES) conducted by the Korea Centers for Disease Control and Prevention between 2010 and 2012. In the fifth KNHANES, the survey was conducted by extracting 20 households randomly from 192 regions with approximately 100,000 people over the age of one. People were divided to three groups: pediatric (one to 11 years old), adolescents (12 to 18 years), and adults (19 years or older). The survey was composed of a health interview, health examination, and a nutrition survey [26]. Data is opened to public and is freely available for research purposes.

**Fig. 1:**
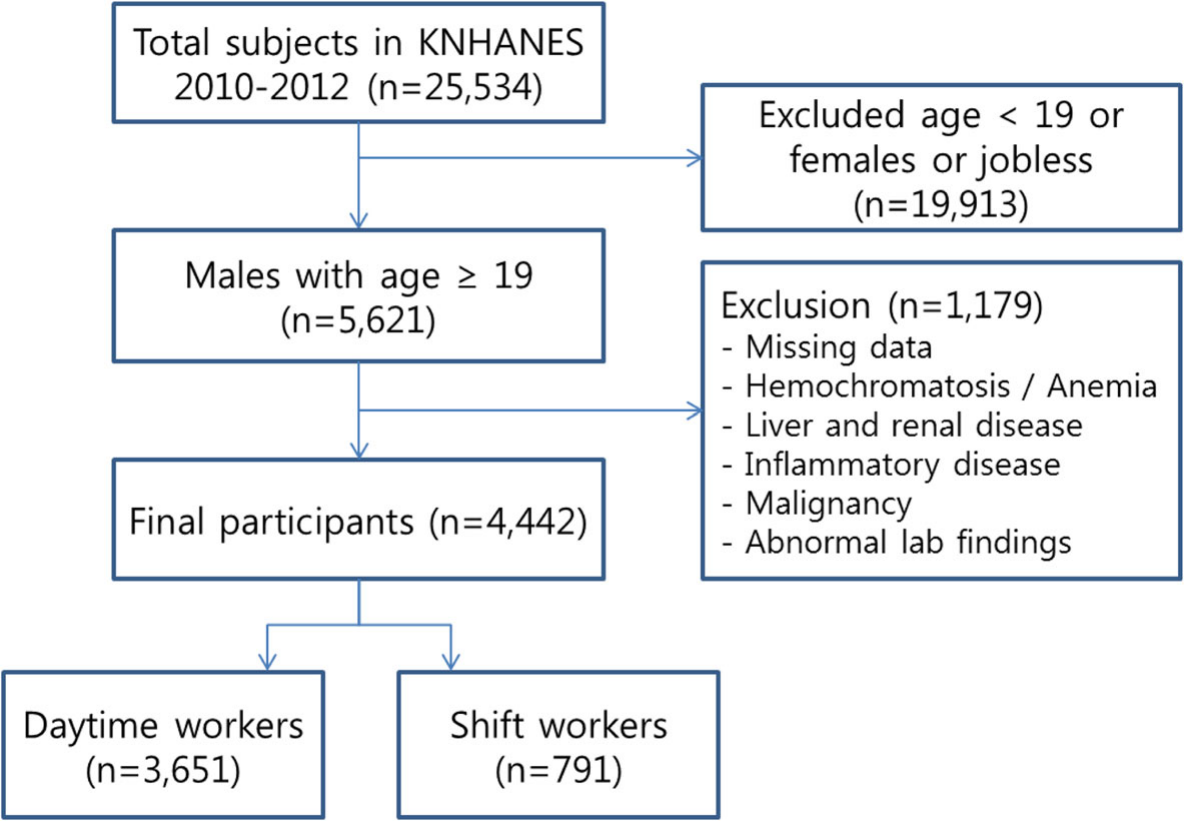
A flow of the study design

The fifth KNHANES had 25,534 initial participants. We included male workers who were over the age of 19 and who included information on the duty hours section of the health interview survey (*n* = 5621). We excluded participants if there was missing data on serum ferritin and blood pressure. We also excluded participants who had inflammatory diseases like arthritis, liver cirrhosis, chronic liver disease, chronic renal disease, malignancy or probable hemochromatosis based on serum ferritin levels (> 300 ng/mL for men) [2, 3]. Some participants were excluded if they had anemia or an iron deficiency states; participants with < 13 g/dL hemoglobin (Hb) or < 10 ng/mL ferritin were not included [27]. Participants with serum creatinine (Cr) > 1.4 mg/dL, serum liver enzymes (either aspartate aminotransferase [AST] or alanine aminotransferase [ALT]) > 80 IU/L, or white blood cell (WBC) counts > 10,000 cells/uL was also excluded from the study. After these exclusions, 4442 participants (3651 daytime workers and 791 shift workers) were included in our final analysis (Fig. 1).

### Data collection

The health interview surveys and health examination surveys were completed at mobile health examination centers. The health examination survey assessed body measurements, blood pressure and pulse measurements, various diagnostic examination and etc. The health interview survey assessed various factors such as working hours, disease morbidity, physical impairment, medication use, smoking and alcohol history. Trained medical investigators performed physical examinations following standardized procedures. Height and body weight were measured respectively to the nearest 0.1 cm and 0.1 kg. For the measurements, participants were asked to remove their shoes and dress in disposable examination gowns. Waist circumference (WC) was measured to the nearest 0.1 cm at the midpoint between the lower border of the rib cage and the iliac crest. Body mass index (BMI) was calculated as the ratio of weight/height^2^ (kg/m^2^). Blood pressure was measured on the right arm in a sitting position using a standard mercury sphygmomanometer. Blood pressure was measured three times, with a 30s rest between each measurement, and the participant’s blood pressure was calculated as the mean blood pressure of the second and third measurements. Blood samples were obtained from the participants from the antecubital veins after an overnight fast. Serum ferritin concentrations were measured using a 1470 WIZARD gamma-Counter with Immunoradiometric Assay (PerkinElmer/Finland). Total cholesterol (TC), triglycerides, fasting blood sugar (FBS), hemoglobin, total iron binding capacity (TIBC), WBC counts, creatinine, vitamin D (VitD) and liver enzymes (AST and ALT) were also recorded [26]. Alcohol consumption was categorized by frequency of drinking; non-drinkers and occasional drinkers were categorized as those who consumed alcohol ≤1 day/month), and regular drinkers were categorized as those who consumed alcohol ≥2 days/month. Smoking status was categorized as current smoker, former smoker, and never smoker.

### Definitions hypertension and working type

Hypertension was defined as a systolic blood pressure (SBP) greater than or equal to 140 mmHg, or a diastolic blood pressure (DBP) greater than or equal to 90 mmHg. Those taking antihypertensive medications were also considered to have hypertension regardless of blood pressure values.

The two working types were classified as “daytime workers” and “shift workers” using the duty hour information collected in the interview. Those who worked from 6 am to 6 pm were consider daytime workers while those who worked from either 2 pm to 12 pm in the evening or 9 pm to 8 am overnight were considered shift workers. Those who indicated that they worked “shift work,” “day and night regular shifts,” “24 hour work shifts,” “split work,” or “irregular shift work” were categorized as shift workers.

### Statistical analysis

Complex sample analyses were used for the KNHANES data for weighting all values following the guidance of statistics from the Korea Centers for Disease Control and Prevention in this study [26]. The data were sorted into quartiles based on serum ferritin concentrations for participants: quartile 1, ≤ 66.82 ng/mL; quartile 2, 66.83–100.78 ng/mL; quartile 3, 100.79–149.13 ng/mL; and quartile 4, > 149.13 ng/mL. General characteristics of participants were derived by a descriptive method for continuous variables and Chi-square test for categorical variables after data weighting. A general linear model was applied to compare continuous variables according to the quartiles of serum ferritin. The interaction of serum ferritin and working type on the prevalence of hypertension was evaluated by logistic regression analysis after adjustment for age, FBS, TC, TIBC, BMI, smoking status and alcohol consumption. In addition, a logistic regression analysis with adjustment was conducted to examine the association between serum ferritin and the prevalence of hypertension after stratifying the working type into daytime workers and shift workers. Data were analyzed using SPSS 20.0 (SPSS, Chicago, IL) to account for the complex sampling design.

## Results

The general and clinical characteristics of the study participants according to the quartile groups of serum ferritin levels are presented in Table 1. Prevalence of hypertension increased as the serum ferritin quartile increased. The TIBC concentrations and age tended to decrease with increasing serum ferritin quartiles. There were no significant differences in VitD and SBP.

**Table 1:** General and clinical characteristics according to the quartiles of serum ferritin

Variables	Quartiles of serum ferritin (ng/mL)				
	Quartile 1 (≤ 66.82)	Quartile 2 (66.83–100.78)	Quartile 3 (100.79–149.13)	Quartile 4 (> 149.13)	P for trend
N	1111	1110	1111	1110	
Age (year)	43.87 (0.512)	42.10 (0.486)	42.34 (0.459)	42.31 (0.443)	0.025
SBP (mm Hg)	119.58 (0.503)	119.15 (0.553)	119.26 (0.545)	120.95 (0.522)	0.083
DBP (mm Hg)	78.96 (0.411)	79.41 (0.422)	79.65 (0.392)	81.58 (0.400)	< 0.001
WC (cm)	82.29 (0.311)	83.97 (0.376)	84.11 (0.338)	86.05 (0.377)	< 0.001
BMI (kg/m^2^)	23.56 (0.106)	24.14 (0.132)	24.20 (0.116)	24.82 (0.141)	< 0.001
FBS (mg/dL)	95.48 (0.613)	96.63 (0.673)	98.33 (0.893)	101.28 (0.878)	< 0.001
TC (mg/dL)	184.81 (1.204)	188.77 (1.180)	191.67 (1.301)	193.25 (1.396)	< 0.001
Triglyceride (mg/dL)	130.68 (3.151)	143.87 (3.559)	155.31 (4.443)	182.03 (6.145)	< 0.001
AST (IU/L)	21.08 (0.245)	21.89 (0.228)	23.00 (0.292)	24.38 (0.305)	< 0.001
ALT (IU/L)	20.72 (0.371)	23.47 (0.433)	24.78 (0.441)	28.70 (0.548)	< 0.001
Hb (g/dL)	15.23 (0.039)	15.45 (0.036)	15.49 (0.033)	15.60 (0.041)	< 0.001
TIBC (μg/dL)	320.41 (1.401)	309.24 (1.316)	306.42 (1.266)	302.56 (1.539)	< 0.001
Cr (mg/dL)	0.95 (0.004)	0.96 (0.005)	0.96 (0.004)	0.96 (0.004)	0.011
WBC (10^3^/uL)	6.20 (0.050)	6.29 (0.054)	6.31 (0.050)	6.43 (0.051)	0.001
VitD (ng/mL)	18.36 (0.249)	18.35 (0.243)	18.45 (0.243)	18.13 (0.234)	0.540
Variables	Quartiles of serum ferritin (ng/mL)				
	Quartile 1 (≤ 66.82)	Quartile 2 (66.83–100.78)	Quartile 3 (100.79–149.13)	Quartile 4 (> 149.13)	*p*-value
Smoking status (%)					
Quit smoker or non-smoker	681 (58.7)	635 (52.5)	586 (46.9)	605 (50.7)	< 0.001
Current smoker	430 (41.3)	473 (47.5)	524 (53.1)	504 (49.3)	
Alcohol intake (%)					
Less than once a month	445 (37.1)	339 (29.5)	307 (28.3)	258 (20.6)	< 0.001
More than twice a month	664 (62.9)	764 (70.5)	797 (71.7)	845 (79.4)	
Hypertension (%)					
Hypertension (−)	770 (74.8)	760 (73.7)	779 (73.0)	723 (68.5)	0.029
Hypertension (+)	341 (25.2)	350 (26.3)	332 (27.0)	387 (31.5)	

Data are presented as the mean (standard deviation), or numbers (percentages)

*SBP* systolic blood pressure, *DBP* diastolic blood pressure, *WC* waist circumference, *BMI* body mass index, *FBS* fasting blood sugar, *TC* total cholesterol, *AST* aspartate transaminase, *ALT* alanine transaminase, *Hb* hemoglobin, *TIBC* total iron-binding capacity, *Cr* creatinine, *WBC* white blood cell, *VitD* vitamin D

Table 2 presents the odds ratio (OR) and 95% confidence interval (CI) for the prevalence of hypertension according to the interaction of serum ferritin and working type after adjustment for age, FBS, TC, TIBC, BMI, alcohol consumption and smoking status. The reference group consisted of daytime workers who were found to have the lowest quartile of baseline serum ferritin levels. Comparing to the reference group which was the lowest quartile of serum ferritin levels, the OR and 95% CI for hypertension of the highest quartile of serum ferritin levels in daytime workers was 1.426 (95% CI 1.066–1.906). In addition, the OR and 95% CI for hypertension were evaluated with the highest quartile of serum ferritin levels in shift workers comparing to the reference group, and the value was 1.696 (95% CI 1.083–2.658).

**Table 2:** Odds ratios (95% CI) for the prevalence of hypertension according to the interaction of serum ferritin and working type

Variables	Odds ratio (95% confidence intervals)^a^	
	Quartile 1 (≤ 66.82)	Quartile 4 (> 149.13)
Daytime workers	1.000 (ref.)	1.426 (1.066–1.906)
Shift workers	1.035 (0.680–1.576)	1.696 (1.083–2.658)

*FBS* fasting blood sugar, *TC* total cholesterol, *TIBC* total iron-binding capacity, *BMI* body mass index

^a^Logistic regression analysis after adjusting for age, FBS, TC, TIBC, BMI, alcohol intake and smoking status

Table 3 shows that OR and 95% CI for the prevalence of hypertension according to the quartile groups of serum ferritin levels in the daytime workers and shift workers. With unadjusted variables, only serum ferritin levels correlated with the prevalence of hypertension. After all variables were adjusted, the adjusted ORs for hypertension comparing the highest quartile of baseline serum ferritin level with the lowest quartile were 1.372 (95% CI 1.027–1.833) in daytime workers, and 2.009 (95% CI 1.042–3.873) in shift workers, respectively.

**Table 3:** Odds ratios (95% CI) for the prevalence of hypertension according to the quartile groups of serum ferritin levels in each daytime workers and shift workers

	Odds ratio (95% confidence intervals)			
Variables	Daytime workers		Shift workers	
	Unadjusted	Adjusted^a^	Unadjusted	Adjusted^a^
Quartiles of ferritin (ng/mL)				
Quartile 1 (≤ 66.82)	Ref.	Ref.	Ref.	Ref.
Quartile 2 (66.83–100.78)	1.037 (0.811–1.327)	1.125 (0.853–1.484)	1.168 (0.690–1.976)	1.364 (0.738–2.518)
Quartile 3 (100.79–149.13)	1.127 (0.865–1.469)	1.274 (0.959–1.691)	0.958 (0.571–1.608)	1.088 (0.582–2.031)
Quartile 4 (> 149.13)	1.297 (1.015–1.657)	1.372 (1.027–1.833)	1.726 (1.015–2.935)	2.009 (1.042–3.873)

*FBS* fasting blood sugar, *TC* total cholesterol, *TIBC* total iron-binding capacity, *BMI* body mass index

^a^Adjusted for age, FBS, TC, TIBC, BMI, alcohol intake and smoking status

## Discussion

This cross-sectional study was conducted to determine the association between serum ferritin level and hypertension according to the type of work in male, using large-scale national data representative of the population. This study showed the risk of having hypertension was greater in shift workers than in daytime workers as serum ferritin levels increased.

Some studies have consistently reported significant associations between hypertension and serum ferritin level in men [15–18]. Two of these studies were cross-sectional studies [15, 17], and others were longitudinal studies [16, 18]. According to a recent study, 7104 healthy Korean men who visited a health examination center were surveyed to assess hypertension incidence from 2005 to 2010. An elevated serum ferritin level was found to be independently associated with the incidental risk for hypertension [18].

There is insufficient evidence to explain the underlying mechanism. There are several possible mechanisms about the association between serum ferritin levels and hypertension. One of which includes the development of atherosclerosis by elevated ferritin levels. Ferritin is a ubiquitous intracellular protein that is the key to controlling iron homeostasis and is a widely used biomarker for the diagnosis of iron deficiency [1–3]. Serum ferritin concentrations reflect not only body iron stores but also systemic inflammation [4–6]. Inadequately elevated body iron as oxidative stress can convert less reactive free radicals to more reactive hydroxyl radicals. Elevated body iron can also cause damage to cellular membranes, lipids, proteins, and deoxyribonucleic acid (DNA) [28]. Elevation of ferritin causes oxidative stress, which leads to inflammation, endothelial damage and consequently atherosclerosis. Atherosclerosis process follows after, and then risk of hypertension can be increased. Experimental studies have shown that hypertension is associated with oxidative stress that can contribute to endothelial dysfunction and leads to BP elevation [29].

Many reports and studies over the past several decades have suggested that the endogenous circadian rhythm can collapse due to the effects of shift work on health and ultimately to the destruction of biological homeostasis [22, 30–32]. In a review article, it was reported that the disruption of the circadian rhythm by shift work can lead to psychosocial, behavioral, and physiological stress, which are associated with an increased risk of cardiovascular diseases such as metabolic syndrome, diabetes, and hypertension [22].

Inflammation has a relationship with prevalent and/or incident hypertension and is also related to the ferritin level, which is also known as a positive inflammatory marker [16]. Furthermore, physiological stress due to circadian disruption among shift workers can aggravate inflammation, which is related to cardiovascular disease [22]. We hypothesized that interactions between shift work and serum ferritin would have an impact on the prevalence of hypertension. Our results showed that the OR for hypertension increased in shift workers with the highest quartile of serum ferritin levels when compared to daytime workers with the lowest quartile of serum ferritin levels as expected. After stratification, the OR for prevalence of hypertension was found to be higher in shift worker than in daytime worker according to ferritin level increasing. These results suggest that serum ferritin and shift work together have an effect on the development of hypertension. Further studies are needed to evaluate the underlying mechanism.

This study has some important strengths. First, this study was conducted using a representative sample of the general South Korean population. In addition, complex sample analyses for weighting instead of simple statistical analyses, and strict quality controls have been applied to the study procedures in KNHANES. Second, to our best knowledge, this is the first study investigating the association of serum ferritin levels and prevalence of hypertension according to the working type.

There are some limitations in this study. First, our study population was composed of only men. The level of serum ferritin can change dramatically before and after menopause. Ferritin levels are generally low in women of childbearing age due to iron lost during menstruation, and then the levels increase after menopause. In addition, serum ferritin concentrations in women are difficult to study because they are affected by many variables such as pregnancy, hormone therapy, and gynecological diseases. Therefore, it is unclear whether our results can be extrapolated to women. Second, our study was conducted cross-sectionally, so it was insufficient to clarify the causative relationship between serum ferritin levels and working type on the development of hypertension. Third, our study is an epidemiological study that used existing data from the National Health and Nutrition Survey and therefore we could not assess all known variables related to our research topic. For example, considering the health effects of shift work, the working duration could be an important variable [19, 20, 33, 34], but the working duration was not applicable because it was not assessed in the survey items.

## Conclusions

In conclusion, this study showed that the prevalence of hypertension in male increased according to incremental increases in ferritin levels, especially in shift workers. In addition, serum ferritin levels can also be used as a subsidiary indicator to evaluate hypertension on high variant blood pressure checks.

## Abbreviations

ALT: Alanine aminotransferase
AST: Aspartate aminotransferase
BMI: Body mass index
CI: Confidence interval
DBP: Diastolic blood pressure
FBS: Fasting blood sugar
Hb: Hemoglobin
KNHANES: Korean National Health and Nutrition Examination Survey
OR: Odds ratio
SBP: Systolic blood pressure
TC: Total cholesterol
TIBC: Total iron binding capacity
VitD: Vitamin D
WBC: White blood cell
WC: Waist circumference
